# A time of transition and tribute at Revista Paulista de Pediatria

**DOI:** 10.1590/1984-0462/2025/43/Editorial

**Published:** 2025-10-20

**Authors:** Clea Rodrigues Leone, Marina Carvalho de Moraes Barros, Tulio Konstantyner, Fabio Carmona

**Affiliations:** a Universidade de São Paulo, Faculdade de Medicina – São Paulo (SP), Brazil.; b Sociedade Paulista de Pediatria, Diretoria de Publicações – São Paulo (SP), Brazil.; c Universidade Federal de São Paulo, Escola Paulista de Medicina – São Paulo (SP), Brazil.; d Universidade de São Paulo, Faculdade de Medicina de Ribeirão Preto – Ribeirão Preto (SP), Brazil.

The *Revista Paulista de Pediatria* (RPPed) is undergoing a remarkable transition. After more than two decades under the leadership of Professor Ruth Guinsburg, the journal now enters a new editorial cycle. It is, therefore, a moment to express, with deep gratitude and admiration, our recognition of the magnitude, depth, and solidity of the work carried out by this exceptional person: professor, researcher, and scientific leader.

Professor Guinsburg assumed the editorship of RPPed at a time when the journal was still taking its early steps and striving to reposition itself within the national academic landscape. It was the early 2000s, and the challenges included consolidating the journal as a publication of excellence under the auspices of the *Sociedade Paulista de Pediatria* (SPSP) while simultaneously adapting to the new editorial and technological demands of an increasingly globalized and competitive scientific environment.

From the very beginning of her tenure, Professor Guinsburg led a careful and systematic restructuring. She reformed the editorial process, strengthened the peer-review system, instituted more rigorous criteria for originality, methodological quality, and scientific relevance, and reinforced the editorial board. With fairness and thoughtfulness in her interactions with authors, she worked tirelessly to ensure that published articles not only reflected the best available scientific evidence but were also relevant and helpful for clinical practice and the professional development of Brazilian pediatricians.

The journal's transformation under her leadership was extraordinary. RPPed achieved progressive indexing in major international databases, including Scopus^®^, Medline/PubMed, and Web of Science. These milestones culminated in remarkable scientometric achievements: in 2024, the journal reached a CiteScore of 2.2 and an impact factor of 2.0, its highest to date, placing RPPed in the second quartile (Q2) in both Scopus and the Journal Citation Reports (JCR) rankings, and among the top pediatric journals in Latin America. For a more detailed account of Professor Guinsburg's editorial journey and legacy, we invite readers to consult the accompanying special article published in this issue: *"*Professor Dr. Ruth Guinsburg: her path and example leading the *Revista Paulista de Pediatria.*"

As she concludes this cycle at the helm of RPPed, Professor Guinsburg leaves us not only a well-established scientific journal indexed in major international databases but also a rare example of academic dedication and institutional commitment. Her leadership has always been discreet, firm, and generous, hallmarks of the great masters. She has guided the journal with the calm conviction of one who knows that science is built quietly, through consistent work, respectful dialog, and attentive listening.

Thus, in unison, we thank Professor Guinsburg for her leadership, her ethical example, and her invaluable contribution to Brazilian pediatrics. Her name will remain inextricably linked to the history of RPPed as a symbol of editorial excellence and scientific rigor. Our gratitude extends not only to her professional excellence but also to the sincere friendship and constant personal support she has offered throughout our journey on the editorial board. We are pleased to announce that she will continue her journey with RPPed, now as Editor Emeritus. Finally, to us, the inheritors of this scientific legacy, remains the responsibility to ensure that RPPed continues on its path of robust growth, academic integrity, and international relevance.

On behalf of the Executive Editors, Associate Editors, and the new Editor-in-Chief,

Clea Rodrigues Leone https://orcid.org/0000-0002-5264-6781

Marina Carvalho de Moraes Barros https://orcid.org/0000-0001-6989-3474

Tulio Konstantyner https://orcid.org/0000-0002-7931-9692

Fabio Carmona https://orcid.org/0000-0001-5743-0325

